# A case series of prophylactic negative pressure wound therapy use with purse-string closure in stoma closure wounds in infants

**DOI:** 10.1186/s40792-024-01818-9

**Published:** 2024-01-17

**Authors:** Yuka Kumata, Daisuke Ishii, Seiya Ishii, Keita Motoki, Naomi Ueno, Ranko Hinooka, Hisayuki Miyagi

**Affiliations:** 1 Division of Pediatric Surgery, Department of Surgery, Asahikawa Medical University, Asahikawa, Japan; 2 Nursing Department, Asahikawa Medical University Hospital, Asahikawa, Japan

**Keywords:** Case series, Pediatric ostomy, Negative Pressure Wound Therapy (NPWT), Surgical Site Infections (SSI), Purse-string closure (PSC), Stoma closure

## Abstract

**Background:**

The study introduces the application of negative pressure wound therapy (NPWT) in pediatric stoma closure, emphasizing the importance of enhancing aesthetics and minimizing surgical site infections (SSI).

**Case presentation:**

The case series involves four infants undergoing non-umbilical stoma closure with a combination of purse-string closure (PSC) and NPWT, focusing on aesthetic outcomes and infection prevention. NPWT was initiated immediately after surgery, and patients were monitored every 3–4 days. Notably, none of the four infants experienced SSI or other complications. The patients adequately tolerated NPWT, with no significant adverse events. Furthermore, Manchester Scar Scale (MSS) was 9 [7–10], and Patient and Observer Scar Assessment Scale (POSAS) (observer) was 12.5 [12–19], POSAS (patient) was 12.5 [11–16] (all median values [minimum–maximum]), indicating that excellent aesthetic outcomes were achieved.

**Discussion:**

We emphasizes the significance of aesthetics in pediatric patients; in addition, our findings demonstrate that four infants who received NPWT combined with PSC achieved superior outcomes that did the most recent four infants who underwent PSC only at our institution. It also addresses the risk of SSI in stoma closure and discusses the pros and potential cons of using NPWT in pediatric cases, underlining the need for further research and the accumulation of additional reports.

**Conclusions:**

This is the inaugural report of prophylactic NPWT for pediatric stoma closure, emphasizing the effectiveness of combining PSC and NPWT for SSI prevention and improved aesthetics. The study calls for additional research and reports on NPWT in pediatric cases to further solidify its benefits in this patient population.

## Background

In adults, the combined use of purse-string closure (PSC) and prophylactic negative pressure wound therapy (NPWT) at ostomy closure has been reported to reduce surgical site infections (SSI) [1, 2] and improve aesthetic outcomes [3], reducing the wound healing period [4]. However, there are currently no reports of such procedures in the pediatric population.

Pediatric ostomy is frequently constructed for conditions like anorectal malformations, Hirschsprung's disease, and neonatal bowel perforations. These cases often require temporary construction, easy management, and closure, with minimal complications and a focus on achieving optimal aesthetics. Additionally, reducing the size and visibility of surgical incisions is essential, as it can contribute to enhancing the Quality of Life (QOL) during the child's subsequent growth and development [5].

Ishii et al. reported that, in the case of umbilical stomas, the post-closure cosmetics were significantly superior compared to non-umbilical stomas [6]. In our practice, we often perform umbilical stomas due to their ease of management and excellent closure cosmetics. However, in cases of poor general conditions, low-positioned umbilicus, umbilical infections, or multiple stomas, the patients were constructed non-umbilical stomas. Therefore, in an effort to enhance the aesthetics and reduce the risk of SSI for non-umbilical stoma closures, we attempted to combine PSC with prophylactic NPWT and report our findings.

## Case presentation

From June 2021 to present, we conducted a case series involving four infant patients who underwent non-umbilical stoma construction. During stoma closure, we utilized PSC and NPWT. The isolated bowel segments were anastomosed at both ends using absorbable sutures.

**Fig. 1:**
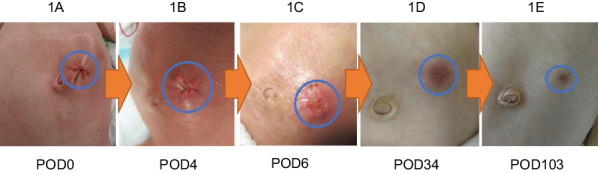
The chronological evolution of the surgical incision. Over time, the wound size decreased, and it developed into a clean scar without any irregularities or indentations in the surrounding skin. **A** Immediate postoperative circular suture; the peritoneum and fascia are closed using absorbable interrupted sutures, followed by a circular skin suture using monofilament absorbable sutures. **B** There is minimal exudate present, but considering sufficient formation of granulation tissue, NPWT is discontinued. **C** Further development of granulation tissue has progressed, and the wound has also flattened out. **D** Epithelialization of the wound has been completed. **E** The wound has minimal irregularities, reduced in size, and has become less conspicuous

**Fig. 2:**
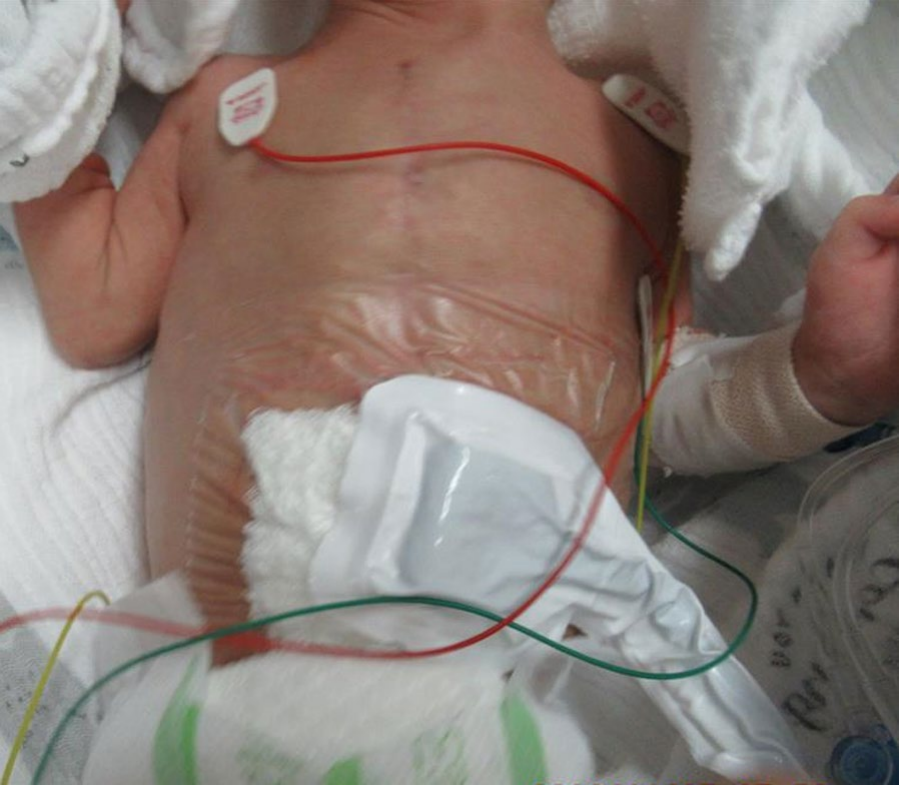
The patient receiving NPWT. NPWT was applied immediately after the surgery

The peritoneum and fascia were closed using absorbable interrupted sutures, followed by a closure of the subcutaneous tissues and dermis around the circumference using monofilament absorbable sutures (Fig. 1A). NPWT was applied immediately after the surgery (Fig. 2) and implemented using the RENASYS® Wound Healing System (Smith and Nephew). Pressure setting and wound filler (cotton or foam) were determined based on the patients' age and body weight. The wound was assessed every 3–4 days, and discontinuation of NPWT was determined based on the absence of wound exudate, in collaboration with a certified Wound, Ostomy, and Continence Nurse. Postoperatively, cefmetazole was administered at a dosage of 80 mg/kg/day for 72–96 h. Approval was obtained from The Asahikawa Medical University Research Ethics Committee (Permit no. 21051). This study was carried out in accordance with the principles of the Declaration of Helsinki.

In these four cases that involved PSC with NPWT after stoma closure, we collected clinical data, including the underlying condition, duration with stoma, operative time at stoma closure, and NPWT settings. The outcomes were evaluated for the occurrence of SSI and complications, and aesthetic appearance. SSI was defined based on the Center for Disease Control guidelines for superficial incisional SSI. Aesthetic assessment was performed using the Manchester Scar Scale (MSS) and Patient and Observer Scar Assessment Scale (POSAS) scores, which were based on medical records and interviews. MSS evaluates parameters such as scar color, surface appearance (matte or shiny), contour, distortion, and texture [7]. POSAS comprises an observer scale (POSAS-O) and a patient scale (POSAS-P), assessing vascularization, pigmentation, thickness, surface roughness, surface area on the observer side, pain, pruritus, color, thickness, relief, and pliability from the patient's perspective [8]. Higher scores on these scales indicate more severe scarring. Both scales are widely used for the quantitative evaluation of aesthetic outcomes in pediatric patients and wound assessment [9, 10].

Demographics are presented in Table 1. The four patients who underwent NPWT included two boys and two girls, with ages ranging from 3.3 months to 4 months. The underlying conditions were as follows: two cases of focal intestinal perforation, two cases of a congenital intestinal obstruction. The intestinal segments included the ileum in all cases. The median operative time of the stoma closure was 82 min (range 64–83 min). Setting of NPWT and results are in Table 2. The median number of dressing changes during the period during NPWT was 0 (range 0–1). There were no cases of postoperative SSI or other complications and no severe adverse events related to NPWT were observed. In all patients, the postoperative course was uneventful. In terms of aesthetic outcomes, MSS was 9 [7–10], POSAS(Observer) was 12.5 [12–19], POSAS(Patient) was 12.5 [11–16] (all median values [minimum–maximum]). The chronological aesthetic evolution of the surgical incision is presented in Fig. 1.

**Table 1:** Demographics

Case	1	2	3	4
Age (months)	4	3.3	3.9	4
Corrected age at SC	44w6d	52w0d	40w2d	54w6d
Sex	F	M	M	F
BW at SC (kg)	2.15	3.16	2.01	4.10
Primary disease	Focal intestinal perforation	Congenital intestinal obstruction	Focal intestinal perforation	Congenital intestinal obstruction
Duration with stoma (days)	110	98	105	118
OT at SC (min)	64	83	81	83

SC: Stoma closure; BW: Body weight; OT: Operative time

**Table 2:** Setting of NPWT and results

Case	1	2	3	4
Dressing changes	0	0	1	1
Pressure setting	− 50 mmHg continuous	− 60 mmHg * 5 min/ − 25 mmHg * 2 min	− 60 mmHg * 5 min/ − 25 mmHg * 2 min	− 70 mmHg * 5 min/ − 25 mmHg * 5 min ↓ − 80 mmHg * 5 min/ − 25 mmHg * 2 min
Filler	Cotton	Cotton	Cotton	Foam → cotton
SSI	–	–	–	–
Complication	–	–	–	–
MSS	10	8	10	7
POSAS(O)	13	12	12	19
POSAS(P)	14	11	11	16

NPWT: negative pressure wound therapy; SSI: surgical site infections; MSS: Manchester Scar Scale; POSAS: Patient and Observer Scar Assessment Scale; O: observer; P: patient

## Discussion

This represents the world's first report of the combined use of PSC and NPWT for pediatric stoma closure wounds. In all four cases, there were no occurrences of SSI or any other complications, affirming the safety and effectiveness of prophylactic NPWT for pediatric stoma closure wounds. Notably, NPWT was found to be highly valuable for improving the aesthetics of surgical wounds, which is crucial for enhancing the QOL of pediatric patients [5]. The aesthetics of surgical wounds in children have been reported to influence their overall well-being. We conducted statistical analysis to determine if there were differences in aesthetic outcomes of non-umbilical stoma closure wounds among the four infants that received NPWT in combination with PSC and the most recent four infants that underwent PSC only at our institution (Table 3). Statistical significance was set at *P* < 0.05. There was no significant difference in surgical background and conditions. Regarding aesthetic outcomes, both MSS and POSAS(P) showed significant differences, indicating superior outcomes in the NPWT group, although no significant difference was observed in POSAS(O). This result may be because of the small sample size; however, we recommend the accumulation of additional cases to ensure a more accurate analysis.

**Table 3:** Statistical analysis between the NPWT group and the only PSC group

	NPWT	Only PSC	*p*-value
*n*	4	4	–
Male:female	4:0	2:2	0.43
Corrected age at SC (days)	336.00	369.50	0.59
Age at SC (months)	3.80	5.88	0.24
BW at SC (kg)	2.86	3.80	0.51
Duration with stoma (days)	107.75	172.50	0.22
OT at SC (min)	77.75	82.25	0.70
MSS	8.75	16.75	0.01
POSAS(O)	14.00	29.25	0.06
POSAS(P)	13.00	28.50	0.03

NPWT: negative pressure wound therapy; PSC: purse-string closure; BW: body weight; SC: stoma closure; OT: operative time; MSS: Manchester Scar Scale; POSAS: Patient and Observer Scar Assessment Scale; O: observer; P: patient

Statistical analysis was conducted using respective mean values. MSS and POSAS(P) showed significant differences, although no significant difference was observed in POSAS(O).

SSIs are known to occur more frequently in procedures involving stoma closure due to the risk of contamination by intestinal contents and its nature of being a secondary surgery [11]. Studies have reported a 17.3% incidence of SSI in pediatric patients who underwent stoma closure using linear skin closure (LSC) [12]. Other studies in low birth weight infants have shown a 37% incidence of SSI when performing stoma closure by LSC [13]. In an effort to reduce the rate of wound infections, the PSC method was proposed by Sutton and colleagues in 2002 [14]. This technique involves closure of the subcutaneous tissues and dermis around the circumference in a purse-string manner, leaving a central aperture of skin open, which provides excellent drainage and reduces the incidence of SSI. Indeed, two systematic reviews and meta-analyses have shown that PSC significantly reduces the risk of SSI compared to traditional LSC [11, 15]. Some reports have also noted improvements in aesthetic outcomes [16]. On the other hand, PSC requires daily wound cleaning and takes longer until complete wound closure [17], which are its disadvantages. To further reduce the incidence of SSI and shorten the healing period, as well as decrease the number of interventions, there have been increasing reports on combining PSC and NPWT in recent years [1–4]. Recent guidelines also recommend the use of prophylactic NPWT to prevent SSI in surgical wounds [18]. However, there are limited reports on the use of prophylactic NPWT in pediatrics [19], and there are currently no reports on its use in pediatric stoma closure wounds.

NPWT promotes wound healing by closing the wound under a negative pressure environment and applying negative pressure drainage. The mechanisms of action for promoting wound healing include ① promoting contraction, ② reducing bacterial load, ③ increasing wound blood flow, ④ removing excess exudate and reducing edema, ⑤ providing physical stimulation to cells and tissues, among others [20].

For prophylactic NPWT use in stoma closure in adults, reports have shown a reduction in SSI [1, 2], improved aesthetics [3], and shorter wound healing periods [4]. In pediatric cases, the prophylactic NPWT on contaminated laparotomy incisions has resulted in significantly better outcomes compared to classic dressings, with improvements in SSI rates, stay periods at hospital and postoperative incision dehiscence [19]. Other advantages of NPWT include the ability to accurately measure fluid loss, enabling strict fluid management [21], as well as the need for fewer dressing changes compared to classic dressings [21]. Particularly in pediatrics, the reduction in the frequency of dressing changes is a significant benefit, as children often experience fear, anxiety, and pain associated with these changes, which is of greater concern compared to adults [22]. On the other hand, a major adverse event following NPWT placement, such as bleeding, is an extremely rare complication in pediatric patients, with a reported incidence of less than 1% [23]. Systemic infections like sepsis are also serious complications following NPWT placement, with reports of sepsis occurring in adult patients after sponge placement [24]. In pediatrics, the risk of sepsis due to bacterial proliferation in the wound is high, as their skin barrier is immature [23]. Nevertheless, a review of pediatric NPWT cases has revealed that the occurrence of sepsis is extremely rare [23].

As previously mentioned, the safety and efficacy of NPWT usage in pediatrics are being demonstrated. However, there is no consensus regarding the settings for suction pressure, foam selection, the duration of usage, and the frequency of dressing changes. The RENASYS® system offers two types of fillers: cotton and form fillers. The choice of a filler was based on factors such as age, weight, and corrected age at stoma closure, which we considered factors that contribute to determining the skin condition. In this study, we selected variable pressure therapy (VPT) mode as the suction mode. Unlike the intermittent mode, the distinctive feature of VPT mode is that it never turns off completely. In many cases of NPWT usage in pediatrics, continuous mode is often employed. Nonetheless, studies targeting adults have reported the superiority of intermittent or VPT modes in terms of granulation tissue formation [24], contraction rates [24], and increased blood flow [25]. On the other hand, the intermittent mode has been criticized for issues such as exudate leakage when reducing the negative pressure and pain during negative pressure application [26]. Owing to these drawbacks of the VPT mode, the continuous mode is the most used mode currently and is believed to be adequately effective. However, VPT mode, which doesn't completely turn off the negative pressure, has been reported to reduce pain compared to the intermittent mode, and it also has a high potential of preventing leakage. In the 4 cases we experienced, pain and leakage were not issues, and although further experience and research are needed, we believe that VPT mode is effective. Furthermore, when configuring NPWT usage in pediatric cases, various factors need to be considered, such as age, gestational age, weight, wound type and size, wound location, skin fragility, responsiveness to treatment, and pain tolerance. To establish guidelines for safe and effective NPWT usage in pediatrics, further research is required, including prospective randomized trials.

## Conclusion

This report is the world's first to present prophylactic NPWT for stoma closure wounds in infants. The use of NPWT in combination with PSC effectively prevents SSI and statistically improves postoperative aesthetics. We intend to continue using NPWT in all cases of non-umbilical stoma closure wounds.

However, the use of NPWT for other abdominal surgical wounds, considering the varying frequency of SSI based on the wound contamination levels would be essential. Expanding the sample size for further investigation seems necessary. Our future research will involve exploring the use of NPWT for primary wound healing. Furthermore, our study is significant, because it establishes the safe and effective use of NPWT for extremely low birth weight infants.

## Data Availability

All data supporting our findings are contained within manuscript.

## Abbreviations

PSC: Purse-string closure
NPWT: Negative pressure wound therapy
SSI: Surgical site infections
MSS: Manchester Scar Scale
POSAS: Patient and Observer Scar Assessment Scale
LSC: Linear skin closure
VPT: Variable pressure therapy

## References

[1] Okuya K, Takemasa I, Tsuruma T, Noda A, Sasaki K, Ueki T (2020). Evaluation of negative-pressure wound therapy for surgical site infections after ileostomy closure in colorectal cancer patients: a prospective multicenter study. Surg Today.

[2] Wierdak M, Pisarska-Adamczyk M, Wysocki M, Major P, Kołodziejska K, Nowakowski M (2021). Prophylactic negative-pressure wound therapy after ileostomy reversal for the prevention of wound healing complications in colorectal cancer patients: a randomized controlled trial. Tech Coloproctol.

[3] Carrano FM, Maroli A, Carvello M, Foppa C, Sacchi M, Crippa J (2021). Negative-pressure wound therapy after stoma reversal in colorectal surgery: a randomized controlled trial. BJS Open..

[4] Sung IK, Kim S (2023). The effectiveness of negative-pressure wound therapy for wound healing after stoma reversal: a randomized control study. Ann Surg Treat Res.

[5] Eichler A, Köhler-Jonas N, Stonawski V, Purbojo A, Moll GH, Heinrich H (2019). Child neurodevelopment and mental health after surgical ventricular septal defect repair: risk and protective factors. Dev Med Child Neurol.

[6] Ishii D, Kumata Y, Ishii S, Motoki K, Miyagi H (2023). Quantitative evaluation of pediatric umbilical loop stomas: 2 decades of experience from a single institution. Pediatr Surg Int.

[7] Beausang E, Floyd H, Dunn KW, Orton CI, Ferguson MW (1998). A new quantitative scale for clinical scar assessment. Plast Reconstr Surg.

[8] Draaijers LJ, Tempelman FR, Botman YA, Tuinebreijer WE, Middelkoop E, Kreis RW (2004). The patient and observer scar assessment scale: a reliable and feasible tool for scar evaluation. Plast Reconstr Surg.

[9] Xu PP, Chang XP, Zhang X, Chi SQ, Cao GQ, Li S (2019). Transumbilical enterostomy for Hirschsprung's disease with a two-stage laparoscopy-assisted pull-through procedure. World J Gastroenterol.

[10] Vercelli S, Ferriero G, Bravini E, Stissi V, Ciceri M, Rossetti S (2017). Cross-cultural adaptation, reproducibility and validation of the Italian version of the Patient and Observer Scar Assessment Scale (POSAS). Int Wound J.

[11] McCartan DP, Burke JP, Walsh SR, Coffey JC (2013). Purse-string approximation is superior to primary skin closure following stoma reversal: a systematic review and meta-analysis. Tech Coloproctol.

[12] Koo EJ, Jung E (2023). Purse-string closure for stoma reversal in pediatric patients. Adv Pediatr Surg.

[13] Sunouchi T, Nishi A, Takazawa S, Maruyama K (2020). The perioperative care and the results of stoma closure operation in low birth weight infants at our children’s medical center. J Jpn Soc Perin Neon Med..

[14] Sutton CD, Williams N, Marshall LJ, Lloyd G, Thomas WM (2002). A technique for wound closure that minimizes sepsis after stoma closure. ANZ J Surg.

[15] Hajibandeh S, Hajibandeh S, Kennedy-Dalby A, Rehman S, Zadeh RA (2018). Purse-string skin closure versus linear skin closure techniques in stoma closure: a comprehensive meta-analysis with trial sequential analysis of randomised trials. Int J Colorectal Dis.

[16] Ortqvist L, Almström M, Ojmyr-Joelsson M, Wigander H, Währner A, Wester T (2011). Cosmetic and functional outcome after stoma site skin closure in children. Pediatr Surg Int.

[17] Camacho-Mauries D, Rodriguez-Díaz JL, Salgado-Nesme N, González QH, Vergara-Fernández O (2013). Randomized clinical trial of intestinal ostomy takedown comparing pursestring wound closure vs conventional closure to eliminate the risk of wound infection. Dis Colon Rectum.

[18] Ban KA, Minei JP, Laronga C, Harbrecht BG, Jensen EH, Fry DE (2017). American college of surgeons and surgical infection society: surgical site infection guidelines, 2016 update. J Am Coll Surg.

[19] Chen B, Hao F, Yang Y, Shang Q, Guo C (2017). Prophylactic vacuum sealing drainage (VSD) in the prevention of postoperative surgical site infections in pediatric patients with contaminated laparotomy incisions. Medicine.

[20] Morykwas MJ, Simpson J, Punger K, Argenta A, Kremers L, Argenta J (2006). Vacuum-assisted closure: state of basic research and physiologic foundation. Plast Reconstr Surg.

[21] Pedrazzi NE, Naiken S, La Scala G (2021). Negative pressure wound therapy in pediatric burn patients: a systematic review. Adv Wound Care.

[22] King A, Stellar JJ, Blevins A, Shah KN (2014). Dressings and products in pediatric wound care. Adv Wound Care.

[23] Santosa KB, Keller M, Olsen MA, Keane AM, Sears ED, Snyder-Warwick AK (2019). Negative-pressure wound therapy in infants and children: a population-based study. J Surg Res.

[24] Beral D, Adair R, Peckham-Cooper A, Tolan D, Botterill I (2009). Chronic wound sepsis due to retained vacuum assisted closure foam. BMJ.

[25] Malmsjö M, Gustafsson L, Lindstedt S, Gesslein B, Ingemansson R (2012). The effects of variable, intermittent, and continuous negative pressure wound therapy, using foam or gauze, on wound contraction, granulation tissue formation, and ingrowth into the wound filler. Eplasty.

[26] Lee KN, Ben-Nakhi M, Park EJ, Hong JP (2015). Cyclic negative pressure wound therapy: an alternative mode to intermittent system. Int Wound J.

